# A Novel Duplex Real-Time Reverse-Transcription PCR Assay for the Detection of Influenza A and the Novel Influenza A(H1N1) Strain

**DOI:** 10.3390/v1031204

**Published:** 2009-12-09

**Authors:** Rebecca J. Rockett, Seweryn Bialasiewicz, David M. Whiley, Cheryl Bletchly, Cassandra E. Faux, Stephen B. Lambert, Graeme R. Nimmo, Michael D. Nissen, Theo P. Sloots

**Affiliations:** 1 Queensland Paediatric Infectious Diseases Laboratory, Sir Albert Sakzewski Virus Research Centre, Queensland Children’s Medical Research Institute, Children’s Health Service District, Queensland, Australia; E-Mails: seweryn@uq.edu.au (S.B.); d.whiley@uq.edu.au (D.M.W.); cassyfaux@uq.edu.au (C.E.F.); sblambert@uq.edu.au (S.B.L.); theniss@uq.edu.au (M.D.N.); t.sloots@uq.edu.au (T.P.S.); 2 Clinical Medical Virology Centre, University of Queensland, Queensland, Australia; 3 Microbiology Division, Pathology Queensland Central, Royal Brisbane and Women’s Hospital Campus, Queensland, Australia; E-Mails: Cheryl_Bletchly@health.qld.gov.au (C.B.); Graeme_Nimmo@health.qld.gov.au (G.R.N.)

**Keywords:** influenza, H1N1, real-time, PCR, detection

## Abstract

Timely implementation of antiviral treatment and other public health based responses are dependent on accurate and rapid diagnosis of the novel pandemic influenza A(H1N1) strain. In this study we developed a duplex real-time PCR (RT-PCR) (dFLU-TM) assay for the simultaneous detection of a broad range of influenza A subtypes and specific detection of the novel H1N1 2009 pandemic strain. The assay was compared to the combined results of two previously described monoplex RT-PCR assays using 183 clinical samples and 10 seasonal influenza A isolates. Overall, the results showed that the dFLU-TM RT-PCR method is suitable for detection of influenza A, including the novel H1N1 pandemic strain, in clinical samples.

## 1. Introduction

Accurate and rapid diagnosis remains an integral part of the public health response as the pandemic novel influenza A(H1N1) virus (swH1N1) continues its global spread. To this effect, real-time reverse-transcription polymerase chain reaction (RT-PCR) methods are now the mainstay of swH1N1 testing, owing to their increased sensitivity and rapid result turnaround times compared to traditional culture-based methods [1–3]. However, a major limitation of RT-PCR methods is sequence variation in primer and probe targets that can lead to false-negative results; this is of particular relevance to emerging viruses such as swH1N1. The influenza A population is comprised of multiple subtypes which show considerable inter and intratypic sequence variation, particularly in the haemagglutinin (HA) and neuraminidase (NA) genes. Recent nucleotide alignments on the Genbank and GISAID databases show that the swH1N1 HA and NA sequences are continuing to accrue point mutations. With many swH1N1 RT-PCR assays targeting these sequences, it is therefore likely that assay sensitivities will ultimately be compromised by continuing mutation. For this reason we have developed a duplex RT-PCR (dFLU-TM) assay using two probes; one for broad range detection of influenza A subtypes (Consensus-FAM) and the other for specific detection of the swH1N1 pandemic strain (swH1N1-VIC). The assay was compared to the combined results of two previously described monoplex real-time PCR assays; a screening WhS1-FluA-5N influenza A RT-PCR that detects the matrix gene of human and animal influenza A subtypes [2] and a swH1-PCR that specifically detects the HA sequence of the pandemic novel H1N1 virus [3].

## 2. Results and Discussion

The results of the dFLU-TM assay gave good agreement with the previously described WhS1-FluA-5N and swH1-PCR assays (Table 1). Briefly, the assay was evaluated using two specimen banks; bank 1 comprised samples collected prior to 2009 and bank 2 comprised positive samples from the 2009 pandemic. Of the 81 clinical samples from bank 1, 45 were positive and 33 were negative by both the dFLU-TM (Consensus-FAM only) and reference assays and 3 samples provided discrepant results, providing a clinical sensitivity of 94% and specificity of 100% for these pre 2009 samples. Of the 102 clinical samples from bank 2, 97 were positive by both the dFLU-TM (Consensus-FAM and swH1N1-VIC probes) and reference assays and 5 samples provided discrepant results, providing a clinical sensitivity of 95% for these 2009 pandemic samples. Ten seasonal H1N1 and H3N2 isolates (years 2000–2008) were tested to further investigate the dFLU-TM assay specificity and all provided positive results in the Consensus-FAM probe and negative results for the swH1N1-VIC component.

Of the eight discrepant results, all produced late cycle threshold (Ct) values, ranging from 36 to 40 cycles (Table 1). Ct values are semi-quantitative markers that are indirectly proportional to viral load, suggesting that these samples contained the lowest quantity of viral genomes observed in the study. Thus, sampling errors or stochastic fluctuations at the assay’s threshold of sensitivity associated with low copy number may account for some of these discrepancies. Additionally, the original samples were unavailable for retesting. Therefore, degradation of nucleic acid as a result of freeze thawing of the extracts may also account for these discrepancies.

Investigation of novel swH1N1 matrix gene sequences on the National Center for Biotechnology Information website showed that the swH1N1-VIC probe target was highly conserved. Of 824 sequences containing the swH1N1-VIC probe target region, 819 provided complete homology with the probe sequence. Only five sequences provided mismatches with the probe. These comprised a single nucleotide substitution in each sequence at four different positions. Our experience suggests that a single mismatch in an MGB probe will reduce fluorescent signal but will not lead to a false-negative result [4].

Overall, the results show that the dFLU-TM is sensitive and specific for detection of influenza A in clinical samples. A notable advantage of the method is that it provides detection of influenza A and simultaneous confirmation of the pandemic novel swH1N1 subtype. Further, the assay targets the highly conserved matrix gene of influenza A, which is more stable than the HA gene targeted by most other swH1N1 RT-PCR methods.

## 3. Experimental Section

Two sample banks were used to test the performance of the dFLU-TM assay. The first bank consisted of 81 clinical specimens (72 Nasopharyngeal aspirates, 6 swabs and 3 bronchial wash specimens) and 10 influenza A clinical isolates (H1N1 n = 4; H3N2 n = 6) collected prior to the swH1N1 pandemic (years 2000–2008). Of the 81 clinical specimens, 48 were positive (H3N2 = 21, H1N1 = 5 and 22 were untyped) and 33 specimens were negative by the influenza A WhS1-FluA-5N PCR (2). The second bank consisted of specimens collected during the novel H1N1 pandemic (July to August 2009) and consisted of 102 samples (17 nasopharyngeal aspirates and 85 swabs) providing positive results for Influenza A. Of these, 100 specimens were positive by the WhS1-FluA-5N PCR and by a PCR specific for the swH1N1 virus (swH1-PCR; reference) and targeting the haemagglutinin gene. The remaining two samples were negative by the WhS1-FluA-5N PCR and positive by the swH1-PCR. Respiratory samples and controls (200 μl) were extracted using the Corbett X-tractor Gene (Corbett Robotics, Australia) and the Corbett DX Xtraction kit (Corbett Robotics, Australia) according to manufacturer’s instructions. Sample extracts were stored at −80 °C prior to testing.

The duplex dFLU-TM assay was based on the WhS1-FluA-5N PCR. Briefly, the dFLU-TM assay was performed as previously described using the consensus primers and “Consensus-FAM” TaqMan probe (2) but with the addition of 0.4 μM of a minor-groove binder “swH1N1-VIC” TaqMan probe (5′vic-ACTGGAAAGTGTCTTTGCAG-mgb3′) for specific detection of swH1N1. This latter probe targets a unique sequence on the swH1N1 matrix gene located between consensus TaqMan probe and reverse primer target sequences. Amplification and detection were performed using the Qiagen one-step RT-PCR kit (Qiagen, Australia) on the Rotorgene 3000 and 6000 instruments (Corbett Robotics, Australia) with the following conditions: initial holds at 50 °C for 20 minutes and 95 °C for 15 minutes followed by 45 cycles at 95 °C for 15 seconds and 60 °C for 1 minute. Consensus TaqMan and swH1N1 TaqMan probe reactions were distinguished using the Rotorgene Green and Yellow detection channels respectively. RNA from an influenza A(H1N1) virus isolate (Auckland, 2009), provided by the Australian World Health Organization Collaborating Centre for Reference and Research on Influenza (Melbourne, Australia), was used as a positive control. Appropriate negative controls were included in each test run.

Sequence conservation of the swH1N1-VIC probe was verified by investigating novel swH1N1 matrix gene sequences on the National Center for Biotechnology Information website (http://www.ncbi.nlm.nih.gov/genomes/FLU/Database/select.cgi; accessed 13 October 2009).

## 4. Conclusions

The ability to simultaneously detect influenza A and identify the pandemic novel H1N1 strain, combined with use of the highly conserved matrix gene target, makes the duplex dFLU-TM assay suitable for routine screening of influenza A in clinical samples.

## Figures and Tables

**Table 1.** Summary of results. Cycle threshold (Ct) values are provided in parenthesis for discrepant samples.

Total no. of. Specimens	*Reference assays:* WhS1-FluA-5N PCR	swH1-PCR	*dFLU-TM:* Consensus-FAM	swH1N1-VIC
*Bank 1: 2000–2008*				
Clinical samples n = 81				
45	POSITIVE	N/A	POSITIVE	Negative
3	POSITIVE (38 – 40)	N/A	Negative	Negative
33	Negative	N/A	Negative	Negative
Isolates n = 10				
10	POSITIVE	N/A	POSITIVE	Negative
*Bank 2: July to August 2009*				
Clinical samples n = 102				
97	POSITIVE	POSITIVE	POSITIVE	POSITIVE
3	POSITIVE (36 – 37)	POSITIVE	Negative	Negative
2	Negative	POSITIVE (37, 39)	Negative	Negative

N/A = not available, these samples were not tested with the swH1-PCR.

## References

[1] Schweiger E, Zadow I, Heckler R, Timm H, Pauli G (2000). Application of fluorogenic PCR assay for typing and subtyping of Influenza Viruses in respiratory samples. J Clin Microbiol.

[2] Whiley DM, Sloots TP (2005). A 5′-nuclease real-time reverse transcriptase-polymerase chain reaction assay for the detection of a broad range of influenza A subtypes, including H5N1. Diagn Microbiol Infect Dis.

[3] Whiley DM, Bialasiewicz S, Bletchly C, Faux CE, Harrow B, Gould AR, Lambert SB, Nimmo GR, Nissen MD, Sloots TP (2009). Detection of novel influenza A(H1N1) virus by real-time RT-PCR. J Clin Virol.

[4] Whiley DM, Sloots TP (2006). Sequence variation can affect the performance of minor groove binder TaqMan probes in viral diagnostic assays. J Clin Virol.

